# The disclosure of potential conflicts of interest among editors and members of editorial boards in leading ethics journals

**DOI:** 10.1186/s41073-025-00181-z

**Published:** 2025-11-21

**Authors:** Clovis Mariano Faggion

**Affiliations:** Department of Periodontology and Operative Dentistry, University Hospital Münster, Waldeyerstraße 30, 48149 Münster, Germany

## Abstract

**Background and Aim:**

The International Committee of Medical Journal Editors (ICMJE) defines a potential conflict of interest (COI) as a situation where professional judgment could be influenced by secondary interests. Competing interests can introduce bias into the peer-review process, making it essential for all participants to declare any potential COIs. While authors are currently required to disclose their COIs, editors and editorial board members are not held to the same standard. This study aimed to evaluate the extent to which editors and editorial board members of ethics journals report their potential competing interests.

**Methods:**

From October 23 to November 1, 2024, 82 ethics journals selected based on their impact factors were assessed, focusing on the disclosure of potential COIs by editors and editorial board members. Journal websites were examined to determine how editors and board members disclose potential COIs. Additionally, publisher websites were assessed for policies guiding these individuals in reporting COIs during peer review.

**Results:**

Only 2% of the journals disclosed potential COIs for their editors, and 13% provided biographical information about editorial members. None of the journals employed a structured reporting approach, such as the ICMJE disclosure form, despite most claiming adherence to ICMJE and COPE guidelines. There was considerable variability in how journals and publishers guided their editors and board members in reporting their own COIs.

**Conclusion:**

The findings indicate that disclosures of potential COIs by editors and editorial board members in leading ethics journals are often inconsistent and insufficient. Increasing transparency in this area could lead to a fairer and more trustworthy peer-review process.

**Supplementary Information:**

The online version contains supplementary material available at 10.1186/s41073-025-00181-z.

## Introduction

The disclosure of potential conflicts of interest (COIs) by researchers submitting manuscripts for publication is a requirement for many scientific journals. The International Committee of Medical Journal Editors (ICMJE) recommends that authors disclose their relationships and activities when submitting manuscripts [1]. Currently, thousands of scientific journals state that they adhere to ICMJE guidelines [2]. Additionally, the Committee on Publication Ethics (COPE) supports editors and publishers by providing guidance on various ethical aspects of the publication process, including COIs [3]. Several prominent publishers, such as Elsevier, Springer, and Wiley, are members of COPE.

By reporting information on authors’ financial and non-financial relationships related to the research subject, published articles provide readers with essential context to interpret potential biases that may have influenced the research and its outcomes. For instance, evidence indicates that between 16.6% and 32.6% of studies published in two major medical journals had at least one author with a COI [4]. These studies were found to be more likely to present positive findings compared to those with authors without COIs. Another study [5] in the field of environmental and occupational health revealed a connection between the financial COIs of organizations involved in the processing, use, or disposal of industrial and commercial products and negative results in studies assessing adverse events in humans.

On the opposite side of the peer-review process, editors and editorial board members are responsible for deciding whether to accept or reject submitted manuscripts. Like authors, these individuals may have potential COIs that could impact the peer-review process [6]. For example, one study [7] analysing over 100,000 articles published in the journal *PLOS One* found that prior co-authorship relationships between the editor handling a submitted manuscript and the authors significantly influenced manuscript handling times, often speeding up editorial decisions compared to other submissions where the editor had no prior relationship with the authors. A recent article [8] reported misconduct by a senior guest editor involving the editorial handling of authorship, stemming from a non-financial COI. Following the submission of a manuscript to a journal’s special issue, the senior guest editor requested that an additional author from their own institution be added to the manuscript. In response, the authors withdrew their submission.

In some cases, a potential COI involving editors may result in the retraction of an article, even when the grounds for retraction are not fully substantiated, as discussed in another article [9]. The authors of the retracted paper contend that their case was not properly investigated and assert that no misconduct or inappropriate application of methods occurred.

The literature also reports instances of potential financial COIs among editors. One study [10] indicated that 210 (29%) of the 716 editorial board members of five leading spine journals reported a potential COI. Similarly, another study [11] assessing 908 physician editors across 15 orthopaedic surgery journals found varying rates of potential COIs among editorial board members based on the “Open Payments” system. Specifically, in the category of those receiving over $10,000 yearly, between 4 and 73% of editors had potential COIs. Additionally, a study [12] evaluating the disclosure of industry funding among editorial boards of surgery journals included 688 U.S.-based physicians out of a total of 1,002 identified editorial board members. Among these 688 board members, 452 (65.7%) received industry payments, raising concerns about potential financial COIs, while only 3.3% of all reviewed physicians provided declarations.

Given the information presented, it is reasonable to argue that editors and editorial board members should disclose any potential COIs to promote a transparent and equitable peer-review process [13]. The publication process should be considered a reciprocal exchange, in which those responsible for evaluating manuscripts are held to the same ethical standards as those submitting them.

Currently, it is unclear whether information regarding potential COIs is disclosed by the editors and editorial board members of ethical journals. It would be valuable to determine whether this information is publicly available. While there are no guidelines or laws mandating the reporting of potential COIs for editors of scientific journals, having this information publicly accessible would enhance credibility in the publication process and the overall integrity of science.

The aim of this study was to evaluate the disclosure of potential COIs of editors and editorial board members on the websites of leading ethics journals.

## Methods

### Research question

The broad research question guiding this study is: “What is the reporting level of COI statements from editors and editorial board members of ethics journals with identified impact factors?”.

### Selection of journals and data extraction

The journals were selected through the Journal Citation Reports (Clarivate, 2023) [14], accessed via the official Clarivate website. Only journals classified by their impact factor were chosen, as this provides a measurable and comparable criterion regarding their influence. The selected journals were categorized under ETHICS and MEDICAL ETHICS in the Journal Citation Reports. The websites of these 82 journals were thoroughly examined for information regarding potential COIs of editors-in-chief, associate editors, and editorial board members. The editorial board typically consists of experts who provide guidance on the development of the journal. Data was extracted directly into a standardized Excel form. The following information was collected:

- name of the journal;
- number of editors and editorial board members;
- reporting of COI statements (YES/NO);
- type of COI statement (standardized form, e.g., the ICMJE disclosure of interest form; informal text);
- 2023 IF data;
- journal citation index;
- whether the information on editors and editorial board members was reported as "biographical" information;
- name of the publisher;
- type of journal (subscription/hybrid; open-access);
- whether the journal follows COPE guidelines;
- whether the journal follows ICMJE guidelines;
- publisher's guidance regarding the declaration of potential COIs for editors and board members.

### Rationale for the assessment

The initial search for information was conducted on the journal's website. All sections, including the “Instructions for Authors,” were scrutinized to determine whether editors and board members reported their potential COIs. The journal's website was also assessed for any guidance on how editors and board members should declare potential COIs during the peer-review process. Additionally, the assessment of statements concerning the guidance for editors and board members was also extended to the publisher's website whenever a link from the journal pointed to it. Guidance from publishers on how editors should report their own COI was also gathered from the publishers' websites.

The accessibility of COI guidance was assessed. A score of 1 was assigned if COI guidance was available on the journal’s website. If not, the next step was to check whether the journal’s website provided a direct link to the publisher’s website with promptly retrievable COI information, which also received a score of 1. However, if the link led to general ethics information without specifically addressing COI for editors or board members, a score of 2 was assigned (indicating an indirect link). If COI information was already available on the journal’s website, the publisher’s website was not searched (marked as not applicable, n.a.). Detailed information on COI coding is provided in the data extraction form (Supplementary File).

## Results

A total of 82 ethics journals with impact factors were selected. The ethics journals included in the sample covered a range of fields, including medicine, business, the environment, information technology, engineering, and agriculture. These journals were published by 26 different publishers, with five publishers responsible for publishing 55 (73%) of the journals (Table 1). The number of editors for each journal varied from 1 to 94, and the number of editorial board members ranged from 0 to 115. More than 70% of the journals in the sample operate under a subscription or hybrid publishing model, while the remaining journals follow a purely open-access model.

**Table 1:** Characteristics of high-ranked ethical journals

Journals´ characteristics	Frequency (%)
**Journal type**	
- Subscription/hybrid	59 (72)
- Open access	23 (28)
**Publisher**	
- Springer Nature	25 (30)
- Taylor and Francis	11 (13)
- Wiley	8 (10)
- SAGE	7 (9)
- Cambridge	4 (5)
- Others	27 (33)
**Follow ICMJE**	
- Yes	53 (65)
- Not reported	29 (35)
**Follow COPE**	
- Yes	67(82)
- Not reported	15(18)
**Median (IQR) JCI**	0.685 (0.34 to 1.04)
**Median (IQR) JIF**	1.3 (0.7 to 2.2)

Only two (2%) of the journals explicitly reported on potential COIs, and this was only applicable to their editors-in-chief. Of these two journals, one had two editors-in-chief, but only one of them provided a statement. Nearly 90% of the journals failed to report biographical information about their editorial board members. When such biographical details were available, they were typically found on personal webpages, university affiliation pages, or the Open Researcher and Contributor ID (ORCID) website. Notably, none of the journals in the sample utilized a structured form to report potential COIs for editors or editorial board members (Table 2).

**Table 2:** Reporting of conflict of interest of editors and editorial board members of high-ranked ethical journals

Editorial board members´ characteristics	Frequency (%)
**Number of editors**	
- Median (IQR)	7 (3.5 to 9)
**Number of editorial board members**	
- Median (IQR)	29 (21 to 40)
**Reporting of COI statement**	
- Yes	2 (2)
- Not reported	80 (98)
**Type of COI**	
- Financial and non-financial	2 (2)
- Not reported	80 (2)
**Biography reported**	
- Yes	11 (13)
- No	71 (87)

Fifty-three (65%) journals reported they follow ICMJE guidelines, and 67 (82%) journals reported they follow the COPE guidelines. Thirty-one (38%) of the journals provided information on their websites regarding guidance for editors and board members, while 40 (49%) journals included a link to the publisher's website where this information could be accessed. However, most of these links were not explicitly labeled as "guidance for editors/board members on declaring their own COIs." Instead, they directed users to general ethical information provided by the publisher. Consequently, in some cases, locating the relevant information required multiple steps. Eleven (13%) of the journals did not provide any guidance information for editors and board members on declaring their own potential COIs. Further details on the data provided by five major publishers in this sample are available in the supplementary file.

## Discussion

The present findings indicate that there is significant potential for improving the reporting of potential COIs among editors and editorial board members across a comprehensive sample of ethics journals. Only two journals provided clear statements regarding potential COIs, and this was limited to their editors-in-chief. Furthermore, the reporting of guidance for editors and board members on disclosing COIs varied widely among journals. Some journals provided detailed statements, while others offered little or no information. In many cases, finding this guidance on publishers' websites required significant effort. Journals typically provide a link to an ethical section from the publisher, but information on COIs related to editors is often not directly included, making it difficult to locate. Additionally, the intended audience for the guidance was sometimes unclear—whether directed at authors, board members, or editors. Some guidance was overly brief and lacked essential details.

While some journals included biographical details about their editors and editorial board members, this format does not adequately allow readers to identify potential COIs, as there is no standardization in how relevant relationships are reported. For instance, biographical information was often linked externally, and many of these links were non-functional (i.e., they led to dead pages and provided no relevant information). Others included links to the websites of the individuals’ affiliated university departments or personal homepages. This lack of consistency forces readers to conduct their own investigative searches to identify potential COIs. It would be beneficial for this information to be readily accessible to readers, preferably with direct links from the names of editors and editorial board members to standardized COI declarations. Additionally, most of the biographical information focused solely on the affiliations and career achievements of the editors and editorial board members, with no details regarding potential financial and non-financial COIs.

### Comparison to other studies

Some studies have assessed the reporting of information on potential COI of editors and editorial board members. For example, in a study [15] including 82 dental journals with associated impact factors, 11 (12%) editors reported COI forms in comparison to 2% of editors of leading ethics journals. In these dental journals, the most frequent method of reporting potential COIs was through an ICMJE disclosure form. In another study [10] assessing the potential COI of editorial board members from five spine journals, almost 50% of editorial board members did not disclose any statement regarding potential COI. The present study presented COI statements only for two editors and no information on COI statements for editorial board members was found on the journals’ websites. A recent study [16] reported that none of the editors overseeing the peer review of 259 medical manuscripts disclosed any potential COIs in the publicly available peer-review reports.

### Improving transparency in reporting conflicts of interest

There have been suggestions for editors and editorial board members to disclose their potential COIs [13, 17]. However, key organizations focused on ethics in research, such as ICMJE and COPE, have not provided clear guidelines on how editors should report their own potential COIs. These organizations generally advise that editors avoid making decisions on manuscripts submitted by relatives or in situations where personal relationships or activities may influence the acceptance or rejection of the manuscript [15]. Unfortunately, they do not specify how editors should make information about potential COIs available to readers and the public.

To address this gap, organizations like ICMJE and COPE could revise their recommendations to require those involved in manuscript decision-making to disclose information that might lead to potential COIs. Additionally, ethics journals could implement a standardized reporting form for editors and editorial board members to disclose their potential COIs. The ICMJE disclosure of interest form could be made accessible on the websites of these journals, with necessary adaptations for non-medical fields. This form should be regularly updated to keep authors and readers informed about any relationships that could affect the publication of studies. In this sample of ethical journals, most reported on their websites that they follow or are members of COPE but rarely specify which recommendations they adhere to. Similarly, when ICMJE guidelines were mentioned, they were typically related to authorship guidance. Furthermore, publishers' recommendations allow editors to determine what constitutes a potential COI, rather than encouraging them to disclose activities and relationships [19] that could influence their decisions when accepting or rejecting a manuscript. In other words, editors should disclose relevant information and allow readers to assess whether it represents a potential COI, rather than selectively reporting what they personally consider to be a COI.

It can be argued that potential COIs are more prevalent in certain types of journals—particularly those that publish research frequently funded by commercial entities, such as pharmaceutical companies—than in ethics-focused journals. However, in my view, all journals, regardless of their focus, should provide clear guidance on COIs and disclose potential conflicts involving their editorial members. While I recognize that journals publishing commercially funded research may be especially vulnerable to financial COIs, it is equally important to acknowledge that non-financial COIs can influence editorial decisions across all types of journals, as demonstrated in references [7] and [8]. Moreover, journals that strive to uphold high ethical standards should lead by example. This includes establishing and publishing transparent policies and disclosing information that may reveal potential COIs among their editorial teams.

### Situations requiring editorial recusal to avoid conflicts of interest

The circumstances under which an editor should refrain from handling and making decisions regarding a submitted manuscript remain a matter of debate. Nevertheless, several situations may give rise to a potential COI for the editor, which can be considered roughly in order of significance:

- Financial interest in the research outcomes: If the editor stands to gain financially from the results of the study, their judgment may be compromised, potentially biasing the review process.
- Close personal or professional relationships with an author: Strong personal connections or significant collaborations could influence the editor’s objectivity, favoring certain authors or undermining impartial evaluation.
- Direct competitive relationship with the authors: Editors in direct competition with the submitting authors may have conflicts that affect their decision-making, whether consciously or unconsciously.
- Affiliation with the same institution as a submitting author: Institutional ties may create a perception or reality of favoritism, challenging the fairness of the review process.

Establishing clear guidelines for editorial recusal in these situations is essential to ensure transparency, fairness, and integrity in the peer-review process. Publishers have suggested recommendations, as outlined in the supplementary file. However, these procedures are typically conducted internally, within the journal’s organizational domain. Making such procedures publicly accessible could enhance accountability and allow authors, reviewers, and readers to better understand how potential conflicts are managed, though the actual impact of public disclosure on bias prevention or trust has yet to be systematically evaluated.

### Strengths and limitations

To the best of my knowledge, this is the first study to evaluate the extent to which ethics journals report information that could indicate potential COIs among their editorial members. The analysis includes journals with the highest impact in the field, which likely represent best practices in ethics publishing.

A potential limitation of this study is the absence of collaborators directly affiliated with the journals, particularly peer reviewers. Although beyond the scope of this article, reviewers also play a critical role in shaping editorial decisions. As key participants in the decision-making process, they too should be required to disclose any potential COIs. However, as the primary focus of this study was on publicly available information from journal websites—and given that reviewers are typically not listed—this limitation does not compromise the validity of the analysis. Nonetheless, a follow-up study could usefully examine how potential COIs among reviewers are addressed and whether any transparency mechanisms are in place to mitigate associated risks.

## Conclusion

The study reveals that transparency regarding the potential COIs of editors and editorial board members in ethics journals remains insufficient. Very few journals provide clear and accessible information on editors' COIs, and most do not offer guidance or standardized processes for reporting such conflicts. Ethical publishing requires that those responsible for editorial decisions disclose their potential COIs openly, mirroring the expectations placed upon authors. The establishment of standardized COI disclosure mechanisms for editors would enhance the integrity and trustworthiness of the scientific publication process. Organizations such as ICMJE, COPE, and major publishers should lead efforts to formalize guidelines ensuring comprehensive disclosure and accessibility of this information. Ethics journals, as custodians of high ethical standards, should serve as exemplars in transparency by making editorial COI declarations publicly available.

## Supplementary Information

Supplementary Material 1.

Supplementary Material 2.

## Data Availability

All data supporting the findings of this study are included in the supplementary file accompanying this article.

## Abbreviations

COIs: Conflicts of interest
ICMJE: International Committee of Medical Journal Editors
COPE: Committee on Publication Ethics
IQR: Interquartile range
ORCID: Open Researcher and Contributor ID
JCI: Journal citation indicator
