# Supplementary material for: The disclosure of potential conflicts of interest among editors and members of editorial boards in leading ethics journals
Source: Res Integr Peer Rev. 2025 Nov 21;10:25. doi: 10.1186/s41073-025-00181-z (PMC12636210; doi:10.1186/s41073-025-00181-z)
Supplement: Supplementary file 2 — Supplementary Material 2. [file 41073_2025_181_MOESM2_ESM.docx]

**Supplementary file**

Table 1. Publishers' recommendations to editors on disclosing potential conflicts of interest

| **Publisher** | **Recommendation for editors** |
| --- | --- |
| Taylor and Francis | “An editor will: Declare to Taylor & Francis/Routledge, on at least an annual basis, any relevant competing interests that may influence, or may be perceived to influence, their decisions as Editor.” (https://editorresources.taylorandfrancis.com/welcome-to-tf/policies-guidelines/editor-code-of-conduct/) |
| Springer | “Editorial Board Members and Editors are required to declare any competing interests and may be excluded from the peer review process if a competing interest exists. In addition, they should exclude themselves from handling manuscripts in cases where there is a competing interest. This may include – but is not limited to – having previously published with one or more of the authors, and sharing the same institution as one or more of the authors. Where an Editor or Editorial Board Member is on the author list we recommend they declare this in the competing interests section on the submitted manuscript. If they are an author or have any other competing interest regarding a specific manuscript, another Editor or member of the Editorial Board will be assigned to assume responsibility for overseeing peer review. These submissions are subject to the exact same review process as any other manuscript. Editorial Board Members are welcome to submit papers to the journal. These submissions are not given any priority over other manuscripts, and Editorial Board Member status has no bearing on editorial consideration.” |
| Wiley | “Editors should disclose any potential interests, both financial and non-financial, that might appear to affect their ability to objectively oversee peer review or undertake decision-making on a given submission. Financial conflicts of interest include funding from various sources such as advertising, government, charitable or philanthropic institutions and relevant financial or employment interests (e.g., patent ownership, stock ownership, consultancies, or speaker’s fees). Non-financial conflicts of interest refer to personal relationships, personal (including competition or potential rivalry), political, or religious interests. A conflict of interest may also be present in situations where an Editor and submitting author have recently performed research together, sit in the same academic department, or have recently co-authored a research article. Editors should recuse themselves from handling manuscripts or adjudicating on specific integrity or ethic concerns where they have any potential or perceived conflicts of interest that may influence their ability to make unbiased editorial decisions or manage an unbiased peer review process. In these situations they should delegate responsibility for manuscript handling to another Editor.” (https://www.wiley.com/en-us/network/publishing/research-publishing/editors/editor-code-of-conduct) |
| SAGE | “What do Editors need to declare?  All Editors are required to declare any conflicts of interest that may impact the peer review and decision-making process. If a conflict arises, an alternative member of the Editorial board must be appointed and the Editor with the conflict must recuse themselves from the decision-making process. These conflicts include financial and non-financial interests listed above.  If the Editor is based at the same institution as the authors or has previously conducted research with them in the last three years, they should recuse themselves from the peer review and decision-making process.  Journal Editors or members of the editorial board submitting their research to their own journals may do so and must declare their involvement with the journal as a conflict of interest. Where applicable, the Journal Editor or Editorial Board member must recuse themselves from the peer review process. The Journal Editor must appoint another member of the Editorial board who will invite two or more subject experts to evaluate the manuscript.  Guest Editors may submit their research to a special issue or special collection they are leading and must declare a conflict of interest upon submission. Where Guest Editors are involved in peer review and final decisions, their submissions will be handled by an alternate member of the editorial board or the Journal Editor. “ (https://us.sagepub.com/en-us/nam/publication-ethics-and-research-integrity-policy-guidelines-for-authors#Declaration%20of%20conflicting%20interests) |
| Cambridge University Press | “Editors are responsible for declaring any competing interests, whether they apply to individual articles or to their position as an editor of this journal, and recusing themselves as appropriate.” (https://www.cambridge.org/core/journals/journal-of-management-and-organization/information/journal-policies/publishing-ethics) |
